# Subjective quality of life among patients with schizophrenia spectrum disorder and patients with major depressive disorder

**DOI:** 10.1186/s12888-019-2248-7

**Published:** 2019-09-02

**Authors:** Xiao Wei Tan, Esmond Seow, Edimansyah Abdin, Swapna Verma, Kang Sim, Siow Ann Chong, Mythily Subramaniam

**Affiliations:** 1 Research Division, Buangkok Green Medical Park, Institute of Mental Health, 10 Buangkok View, Singapore, 539747 Singapore; 2 Department of Psychosis, Institute of Mental Health, 10 Buangkok View, Singapore, 539747 Singapore; 3 Duke-NUS Medical School, National University of Singapore, Singapore, 169857 Singapore; 4 Lee Kong Chian School of Medicine, Nanyang Technological University, Singapore, 639798 Singapore

**Keywords:** Subjective quality of life, SF-36, Schizophrenia spectrum disorder, Major depressive disorder, Psychiatric symptoms

## Abstract

**Background:**

The goal of clinicians and healthcare workers providing treatment to patients with psychiatric disorders, has shifted over time from focusing on the symptoms alone towards functional improvement. In this study, we aimed to compare the subjective quality of life (QoL) among patients with schizophrenia spectrum disorders and major depressive disorder (MDD).

**Methods:**

QoL scores were collected using 36-item Short Form Survey Instrument. QoL scores were compared between 203 outpatients with schizophrenia spectrum disorders and 185 outpatients with MDD using analysis of covariance. The Positive and Negative Syndrome Scale was administered to assess the severity of psychiatric symptoms among patients with schizophrenia and Personal Health Questionnaire-8 items was utilized to assess the severity of depressive symptoms among patients with MDD. The correlation coefficient (r) of socio-demographic factors and core psychiatric symptoms with QoL were analyzed using multiple linear regression.

**Results:**

As compared to patients with MDD, patients with schizophrenia reported better health scores in all QoL subdomains, except for physical function (PF). Among patients with schizophrenia, old age was correlated with better mental health (MH, *r* = 0.35) and PF (*r* = 0.37). Compared to those of Chinese ethnicity, those of Malay, Indian and other ethnicity were correlated with worse PF (*r* = − 0.43 for Malays; *r* = − 0.30 for Indians and *r* = − 0.34 for other ethnicities). Longer duration of mental illness was correlated with worse MH (*r* = − 0.30), worse PF (*r* = − 0.38) and worse scores on role limitations due to physical health problems (RP, *r* = − 0.30). Among patients with MDD, older age was correlated with worse PF (*r* = − 0.33) and patients without comorbid physical illness reported less bodily pain (*r* = 0.45) and better general health (*r* = 0.34). Moreover, all psychiatric symptoms among patients with schizophrenia were negatively correlated with QoL, but the strength of the correlation was less than that between depressive symptoms and QoL among patients with MDD.

**Conclusion:**

Patients with schizophrenia generally reported better QoL as compared to patients with MDD. The correlates of QoL differed between patients with schizophrenia and patients with MDD. This study adds to the understanding of QoL among patients with mental illnesses and may aid in better management of these patients with different psychiatric diagnoses.

## Background

Schizophrenia spectrum disorders and major depressive disorder (MDD) are severe mental illnesses that affect major life domains, such as mental and psychosocial functioning [1, 2]. The current long-term treatment strategies remain suboptimal for patients with schizophrenia and MDD. A sub-group of them may not respond satisfactorily to drug therapy and experience symptom relapses over a prolonged period of their life [3, 4]. Therefore, the goal of clinicians and healthcare workers has shifted over time from focusing on the psychiatric symptoms alone towards functional improvement.

Both schizophrenia and MDD can affect the patient’s overall Quality of life (QoL). Younger age, female gender, being married and lower education level are important sociodemographic factors associated with low QoL in patients with schizophrenia [5]. Other than socio-demographics, psychotic and depressive symptoms are consistently and negatively associated with QoL domains such as psychological health and social relationships [6–9]. For patients with schizophrenia, positive symptoms such as hallucinations and delusions can cause patients to lose touch with reality and impair their daily functioning [10–12]. Negative symptoms tend to persist longer than positive symptoms and patients who exhibit significant negative symptoms have particularly poorer functioning in both mental and physical activities [13–16]. Depressive symptoms commonly occur in patients with schizophrenia, with a reported prevalence of 30 to 75% [17]. The comorbid depressive symptoms in patients with schizophrenia have often been associated with impaired mental functioning [18–20], poorer subjective QoL [21, 22], higher rates of relapse or re-hospitalization [20, 23, 24] and suicidal ideas [25–28]. Patients with MDD suffer from persistent sadness, low self-esteem and loss of interest. MDD has been shown to account for severely impaired social ability [29], work ability [30], physical functioning and emotional functioning [31]. Patients with MDD were less likely to complete high school or college [32], more likely to experience divorce [33] and more likely to report poor quality of interpersonal relationships [34] than subjects without MDD.

Substantial evidence has shown the association between socio-demographic factors and clinical symptoms with subjective QoL among patients with schizophrenia and MDD. However, due to the wide variations in measurement instruments, study strategies and definitions of QoL, the evidence of direct comparison of QoL among patients with schizophrenia and MDD is still scarce. In this study, we aimed to compare the illness-specific profiles of subjective QoL among patients with schizophrenia and MDD. Furthermore, we were interested in comparing the correlation strength of the socio-demographic factors and core psychiatric symptoms with patient’s subjective QoL. A better understanding of the illness-specific profiles of subjective QoL and the associations between patients’ socio-demographics, lifestyle factors and clinical symptoms can improve the management of patients with mental illnesses through earlier identification of cases needing greater attention.

## Methods

### Study design and participants

This was a single center cross-sectional study. Overall, 251 outpatients meeting primary Diagnostic and Statistical Manual of Mental disorders, Fourth edition (*DSM-IV*) criteria for schizophrenia spectrum disorder and 249 outpatients with a primary diagnosis of MDD were enrolled into this study. Inclusion criteria comprised: (1) being 21–65 years of age and able to provide consent; (2) clinically diagnosed with a primary schizophrenia spectrum disorder or MDD by consulting psychiatrists; (3) literate in the English language, i.e. being able to read and understand English and self-complete the survey form in English. Patients with a history of dementia or intellectual disability were excluded. Ethical approval to conduct the study was obtained from the National Healthcare Group Domain Specific Review Board, Singapore. All participants provided written informed consent before participating in the study. We conducted the interviews at the outpatient clinic. Posters were placed in the clinic to inform attending patients of the ongoing study with information on eligibility criteria and researchers’ contact. All participants were referred by treating clinicians. While researchers administering the questionnaire were not involved in the treatment of these patients, if patients expressed any distress due to the study procedures or expressed any thoughts of self-harm or an impending crisis, the treating clinician was informed of the same.

### Measures

Participants completed several questionnaires which assessed their socio-demographic details including age, gender, religion, years of schooling, marital status, number of children, living conditions, employment status, income, smoking habits, symptom severity and comorbid physical illness,. For employment status, patients who were working full-time/part-time, students and on national service were grouped as “employed”. While patients who were housewife/husband, retired, unemployed but able to work and unemployed due to disability and medical condition were grouped as “unemployed”. Patients’ clinical characteristics, including medical diagnosis, age of diagnosis and medications were extracted from medical records. Duration of mental illness was calculated from age of diagnosis till the year of participation in the current study.

The Positive and Negative Syndrome Scale (PANSS) comprises 30 items to measure the severity of psychopathology in schizophrenia [35, 36]. We adapted the five-factor structure of PANSS, namely positive, negative, cognitive, excitement and depression factors, based on a published factor analysis, which has been validated among patients with early symptoms of schizophrenia spectrum disorder in Singapore [37, 38].

The eight-item Patient Health Questionnaire (PHQ-8) was utilized to assess the severity of depressive symptoms among patients with MDD during the last 2 weeks. The PHQ-8 is comparable with PHQ-9 and is established as a valid diagnostic and severity measure for depressive disorders in large clinical studies as well as epidemiological population-based studies [39]. The scores of each item of PHQ-8 are summed to produce a total score between 0 and 24 points. Higher total scores indicate more severe depressive symptoms. If a response to any of the 8 questions was missing, a total score was not calculated.

The 36-item Short Form Survey Instrument (SF-36) was administered to all patients to measure their subjective QoL during the past 4 weeks. The SF-36 is a patient-reported measure of health status [40], and has been validated in Singapore [41]. The SF-36 consists of eight subscale scores, including general mental health (MH), role limitations due to personal or emotional problems (RE), energy/fatigue or vitality (VT), social functioning (SF), physical functioning (PF), role limitations due to physical health problems (RP), bodily pain (BP) and general health perceptions (GH). Each scale is directly transformed into a 0–100 scale on the assumption that each question carries equal weight. Lower scores indicate worse QoL.

### Statistical analysis

To homogenize the study population, only patients with no records of secondary psychiatric diagnoses (203 outpatients with schizophrenia and 185 outpatients with MDD) were included into this study. All statistical analyses were performed with SPSS (*IBM, v.25*). We used descriptive statistics to establish the socio-demographic and clinical characteristics of the patient sample. Continuous variables were presented as mean ± standard deviation (SD) while categorical variables were presented as count (percentage, %).

Comparison analysis was conducted with *t* tests for continuous variables or chi-square tests for categorical variables to determine the difference of participants’ socio-demographic and clinical characteristics. Differences in QoL subdomain scores between patients with schizophrenia and patients with MDD were compared with analysis of covariance (*ANCOVA*). Using this approach, we evaluated whether the mean scores of subdomains of QoL were equal among patients with a diagnosis of schizophrenia or MDD, while statistically controlling for the effects of other parameters including participants’ socio-demographics and clinical characteristics. Associations of socio-demographic factors and symptom severity with subjective QoL were analyzed by multiple linear regressions. Firstly, several assumptions of multiple linear regressions were tested. There was no multicollinearity among the independent variables including socio-demographics and psychiatric symptoms scores and the variances of error terms were similar across the values of the independent variables. The correlation coefficients (r) were then calculated to compare the strength of correlation between socio-demographics/psychiatric symptoms and QoL subdomain score. Correlation coefficient was converted from the root square of Partial eta-squared (*Ƞ*^*2*^), which was derived from multiple linear regression models. [42] The absolute value of correlation coefficient, was considered as small/weak when 0.1 < *r* < 0.3; was considered as medium/moderate when 0.3 ≤ *r* < 0.5 and was considered as large/strong when *r* ≥ 0.5 [42]. *P* ≤ 0.05 was accepted as significant for all tests.

## Results

Table 1 shows the socio-demographic and clinical characteristics of the patients. About 44.8% of the participants with schizophrenia were females while 48.1% of the participants with MDD were females. Compared to patients with schizophrenia, patients with MDD were younger (37.2 ± 11.0 vs 40.0 ± 10.1 years, *p* = 0.009), had longer years of education (12.8 ± 3.7 vs 11.8 ± 4.2 years, *p* = 0.014), with later age of diagnosis (31.1 ± 10.5 vs 25.8 ± 9.1 years old, *p* < 0.001), shorter duration of mental illness (6.0 ± 5.9 vs 14.4 ± 10.7 years, *p* < 0.001) and fewer were currently on psychotropic medications (85.4% vs 97.0%, *p* < 0.001). Moreover, compared to patients with schizophrenia, more patients in the MDD group were married or divorced (28.1% vs 13.8% were married and 17.8% vs 7.4% were divorced respectively, *p* < 0.001), had children (37.8% vs 16.7%, *p* < 0.001), were living in private housing (13.0% vs 2.5%, *p* < 0.001), were employed (65.9% vs 52.2%) and had higher income (34.0% vs 10.4% ≥2000 SGD/month, *p* < 0.001).

**Table 1:** Patients’ socio-demographic and clinical characteristics

Patient characteristics	Schizophrenia (*n* = 203)	MDD (*n* = 185)	*p* value
Age (years)	40.0 ± 10.1	37.2 ± 11	0.009
Gender			
Female	91 (44.8%)	89 (48.1%)	0.518
Ethnicity			0.267
Chinese	117 (57.9%)	124 (67%)	–
Malay	41 (20.2%)	27 (14.6%)	–
Indian	34 (16.7%)	27 (14.6%)	–
Others	11 (5.4%)	7 (3.8%)	–
Religion			0.475
Christine	67 (33.0%)	59 (31.9%)	–
Buddhism	36 (17.7%)	38 (20.5%)	–
Islam	56 (27.6%)	34 (18.4%)	–
Hinduism	17 (8.4%)	12 (6.5%)	–
Taoism	6 (3.0%)	4 (2.2%)	–
Years of schooling	11.8 ± 4.2	12.8 ± 3.7	0.014
Marital status			< 0.001
Single	151 (74.4%)	92 (49.7%)	–
Married	28 (13.8%)	52 (28.1%)	–
Separated	7 (3.4%)	4 (2.2%)	–
Divorced	15 (7.4%)	33 (17.8%)	–
Widowed	2 (1.0%)	4 (2.2%)	–
With children	34 (16.7%)	70 (37.8%)	< 0.001
Residence			< 0.001
Economic house	170 (83.7%)	152 (82.2%)	–
Private house	5 (2.5%)	24 (13.0%)	–
Others	28 (13.8%)	9 (4.9%)	–
Employment status			< 0.001
Employed	106 (52.2%)	122 (65.9%)	–
Unemployed	97 (47.8%)	63 (34.1%)	–
Income^a^ (SGD/month)			< 0.001
< 2000	106 (52.2%)	71 (38.4%)	–
≥2000	21 (10.4%)	63 (34.0%)	–
Age of diagnosis^a^ (years)	25.8 ± 9.1	31.1 ± 10.5	< 0.001
Comorbid physical disease			
Yes	52 (25.6%)	46 (24.9%)	0.805
Psychotropic medication			
Yes	197 (97.0%)	158 (85.4%)	< 0.001
Duration of mental disease (years)	14.4 ± 10.7	6.0 ± 5.9	< 0.001
Smoking status^a^			0.267
Ex-smokers	23 (11.3%)	16 (8.6%)	–
Never smokers	113 (55.7%)	96 (51.9%)	–
Rare	15 (7.4%)	24 (13.0%)	–
Current smokers	52 (25.9%)	48 (25.9%)	–

^a^Data missing for some participants

We then compared the mean scores of subdomains of QoL among patients with schizophrenia and MDD. After controlling for socio-demographic and clinical characteristics, compared to patients with MDD, patients with schizophrenia reported significantly better scores for both mental well-being subscales (MH, 40.8 ± 5.0 vs 31.0 ± 4.5, *p* < 0.001; RE, 45.5 ± 5.4 vs 38.2 ± 4.9, *p* = 0.002; VT, 52.1 ± 4.8 vs 41.3 ± 4.3, *p* < 0.001; SF, 42.9 ± 4.8 vs 32.7 ± 4.3, *p* < 0.001) and physical well-being subscales (RP, 51.5 ± 4.3 vs 47.4 ± 3.9, *p* = 0.026; BP, 50.6 ± 4.7 vs 45.2 ± 4.2, *p* = 0.006; GH, 56.9 ± 4.6 vs 47.0 ± 4.1, *p* < 0.001) (Table 2).

**Table 2:** Comparison of the mean score of quality of life (QoL) among patients with schizophrenia and patients with major depressive disorder (MDD)

QoL subdomain	Crude			Adjusted^a^		
	Schizophrenia disorder (*n* = 203)	MDD (*n* = 185)	*p* value	Schizophrenia disorder (*n* = 203)	MDD (*n* = 185)	*p* value
MH	46.3 ± 0.8	34.7 ± 0.8	< 0.001	40.8 ± 5.0	31.0 ± 4.5	< 0.001
RE	42.5 ± 0.9	35.8 ± 0.9	< 0.001	45.5 ± 5.4	38.2 ± 4.9	0.002
VT	51.5 ± 0.7	40.0 ± 0.8	< 0.001	52.1 ± 4.8	41.3 ± 4.3	< 0.001
SF	45.0 ± 0.8	35.9 ± 0.8	< 0.001	42.9 ± 4.8	32.7 ± 4.3	< 0.001
PF	48.4 ± 0.7	48.3 ± 0.7	0.899	46.4 ± 4.0	45.6 ± 3.6	0.620
RP	44.4 ± 0.7	44.0 ± 0.7	0.738	51.5 ± 4.3	47.4 ± 3.9	0.026
BP	50.6 ± 0.8	46.9 ± 0.8	0.001	50.6 ± 4.7	45.2 ± 4.2	0.006
GH	49.3 ± 0.7	40.7 ± 0.8	< 0.001	56.9 ± 4.6	47.0 ± 4.1	< 0.001

^a^Adjusted for age, gender, race, religion, school years, marital status, children, residence, employment status, income, comorbid physical disease, currently taking medicine, duration of mental disease, smoking status and age of onset of mental illness

Correlation strength of patients’ socio-demographics with subdomain score of QoL was calculated. Among patients with schizophrenia, older age was correlated with better MH (*r* = 0.35) and PF (*r* = 0.37) with a moderate strength. Compared to Chinese ethnicity, Malays, Indians and other ethnicities were correlated with worse PF (*r* = − 0.43 for Malays; *r* = − 0.30 for Indians and *r* = − 0.34 for other races). Longer duration of mental illness was correlated with worse MH (*r* = − 0.30), PF (*r* = − 0.38) and RP (*r* = − 0.30). Among patients with MDD, older age was correlated with worse PF (*r* = − 0.33) and patients without comorbid physical illness reported less bodily pain (*r* = 0.45) and better general health (*r* = 0.34) (Table 3).

**Table 3:** Correlation strength of patient’s socio-demographics or clinical characteristics with Quality of life (QoL) subscale score among patients with schizophrenia or major depressive disorder (MDD)

Patient characteristics	MH	RE	VT	SF	PF	RP	BP	GH
Schizophrenia								
Age	0.35^*,a^	0.21	0.09	0.02	0.37^*,a^	0.16	−0.10	0.19
Female vs male	−0.07	− 0.11	− 0.11	− 0.11	0.01	0.11	0.00	0.02
Malay vs Chinese	−0.26^*^	− 0.23^*^	0.06	− 0.18	− 0.43^*,a^	− 0.07	−0.14	0.01
Indian vs Chinese	−0.21	− 0.12	0.15	− 0.05	− 0.30^*,a^	− 0.05	− 0.14	− 0.02
Others vs Chinese	− 0.21	− 0.15	0.11	− 0.11	− 0.34^*,a^	−0.06	− 0.05	−0.04
Buddhism vs Christianity	0.00	0.01	−0.02	− 0.01	− 0.04	−0.09	− 0.01	−0.12
Islam vs Christianity	0.21	0.14	−0.13	0.05	0.32^*,a^	−0.07	0.04	0.11
Hinduism vs Christianity	0.03	0.06	−0.24^*^	− 0.03	0.24^*^	0.02	0.00	−0.03
Taoism vs Christianity	0.08	0.05	0.06	−0.01	− 0.12	− 0.14	0.00	0.05
School years	0.05	0.02	0.17	0.12	0.20	0.20	0.04	0.02
Married vs single	0.06	0.00	0.05	0.10	−0.18	0.20	−0.04	0.02
Separated vs single	0.01	0.08	0.15	0.04	−0.25^*^	− 0.02	−0.10	0.01
Divorced vs single	−0.11	−0.16	− 0.06	0.04	− 0.01	0.12	0.05	0.01
Widowed vs single	N/A	N/A	N/A	N/A	N/A	N/A	N/A	N/A
No children vs with children	0.02	0.07	0.05	0.03	0.00	0.15	−0.09	0.01
Economic house vs private house	0.02	0.24^*^	−0.05	− 0.16	0.13	0.09	0.00	−0.01
Unemployed vs employed	− 0.15	− 0.17	− 0.04	− 0.14	− 0.06	− 0.06	− 0.10	− 0.02
High income vs low income	− 0.06	− 0.03	− 0.02	− 0.04	− 0.06	0.06	0.05	−0.03
Without comorbid physical illness vs with comorbid physical illness	−0.08	− 0.09	0.03	− 0.01	0.08	0.03	0.02	0.13
Without taking medicine vs with taking medicine	−0.08	− 0.01	0.16	− 0.18	− 0.24^*^	− 0.24^*^	− 0.07	− 0.08
Never smoker vs ex-smoker	0.04	0.11	− 0.01	0.15	0.07	0.11	0.19	−0.02
Current smoker vs ex-smoker	0.16	0.01	0.16	0.10	0.08	0.15	0.19	0.02
Duration of mental illness	−0.30^*,a^	−0.26^*^	− 0.09	0.01	−0.38^*,a^	− 0.30^*,a^	0.06	− 0.21
MDD								
Age	0.00	0.07	0.06	−0.03	−0.33^*,a^	−0.12	− 0.14	0.11
Female vs male	−0.26^*^	−0.02	− 0.27^*^	−0.08	− 0.04	−0.06	− 0.01	−0.15
Malay vs Chinese	0.10	0.19	0.08	0.11	−0.08	0.13	0.04	−0.04
Indian vs Chinese	0.18	0.09	−0.01	0.03	−0.09	0.03	−0.07	−0.05
Others vs Chinese	−0.03	0.05	0.02	−0.09	0.04	0.16	−0.10	0.06
Buddhism vs Christianity	0.08	0.05	−0.05	0.15	0.03	0.23^*,a^	0.07	0.01
Islam vs Christianity	0.09	−0.20	0.03	−0.07	0.10	−0.11	0.01	0.18
Hinduism vs Christianity	−0.17	0.08	0.10	−0.07	−0.02	− 0.06	−0.04	0.12
Taoism vs Christianity	−0.07	0.24^*,a^	−0.13	0.11	0.04	0.16	0.19	0.04
School years	0.21	0.04	−0.04	0.14	0.02	−0.05	−0.07	− 0.17
Married vs single	−0.04	− 0.14	0.12	− 0.16	−0.07	− 0.08	−0.05	− 0.05
Separated vs single	0.15	0.06	0.00	−0.01	0.03	0.15	0.17	0.02
Divorced vs single	0.03	0.02	0.17	0.07	−0.17	−0.08	−0.06	0.03
Widowed vs single	0.04	−0.02	0.08	−0.06	−0.07	− 0.04	0.11	− 0.08
No children vs with children	0.00	0.01	0.03	−0.11	−0.16	0.05	0.00	0.07
Economic house vs private house	−0.09	−0.08	− 0.03	0.04	0.09	0.13	−0.02	−0.14
Unemployed vs employed	0.05	−0.11	0.07	0.03	0.10	0.12	−0.02	0.08
High income vs low income	−0.05	0.07	0.00	−0.12	0.28^*^	0.23^*^	0.12	0.16
Without comorbid physical illness vs with comorbid physical illness	0.13	0.12	0.19	0.15	0.25^*^	0.01	0.45^*^	0.34^*^
Without taking medicine vs with taking medicine	−0.05	0.16	0.02	0.27^*^	0.14	0.19	−0.13	0.06
Never smoker vs ex-smoker	0.02	−0.06	0.07	−0.10	0.01	0.03	−0.06	0.00
Current smoker vs ex-smoker	−0.21	−0.11	0.01	−0.05	− 0.08	−0.03	− 0.09	−0.13
Duration of mental illness	0.29^*^	0.05	0.16	0.11	−0.11	−0.12	− 0.16	−0.01

*N/A* Not available

^*^P < 0.05

^a^Absolute value of correlation coefficient 0.30 ≤ r < 0.50

Finally, we analyzed the correlation strength of the severity of patients’ psychiatric symptoms with subdomain score of QoL. Core psychiatric symptoms among patients with schizophrenia spectrum disorders were negatively correlated with all subdomains of QoL with a mild to moderate strength. Among them, higher positive factor score was correlated with more impaired SF (*r* = − 0.31) and higher depressive factor score was correlated with more impaired MH (*r* = − 0.45), VT (*r* = − 0.46) and GH (*r* = − 0.38). Among patients with MDD, higher depressive symptoms score correlated with all subdomains of impaired QoL with moderate to strong strength (MH, *r* = − 0.80; RE, *r* = − 0.67, VT, *r* = − 0.70; SF, *r* = − 0.73; PF, *r* = − 0.42; RP, *r* = − 0.60; BP, *r* = − 0.56; GH, *r* = − 0.57) (Table 4).

**Table 4:** Correlation of psychiatric symptoms with Quality of life (QoL) subscales among patients with schizophrenia or major depressive disorder (MDD)

Clinical symptoms	MH	RE	VT	SF	PF	RP	BP	GH
Schizophrenia^a^								
Positive factor	−0.23^*^	− 0.19	−0.02	− 0.31^*,b^	−0.12	− 0.26^*^	−0.27^*^	− 0.01
Negative factor	−0.18	− 0.17	−0.15	− 0.01	−0.06	− 0.06	−0.03	− 0.03
Excitement factor	−0.08	0.00	−0.09	− 0.08	−0.05	− 0.11	−0.17	− 0.14
Depression factor	−0.45^*,b^	− 0.18	−0.46^*,b^	− 0.06	−0.11	− 0.07	−0.10	− 0.38^*,b^
Cognitive factor	−0.01	− 0.19	−0.04	− 0.06	−0.01	− 0.04	−0.01	− 0.07
MDD^#^								
Clinical symptoms	MH	RE	VT	SF	PF	RP	BP	GH
Depressive symptoms	−0.80^*,c^	−0.67^*,c^	−0.70^*,c^	− 0.73^*,c^	−0.42^*,b^	− 0.60^*,c^	−0.56^*,c^	− 0.57^*,c^

^a^Adjusted for age, gender, race, religion, school years, marital status, children, residence, employment status, income, comorbid physical disease, currently taking medicine, duration of mental disease, smoking status and age of onset of mental illness

^*^P < 0.05

^b^Absolute value of correlation coefficient 0.30 ≤ r < 0.50

^c^Absolute value of correlation coefficient r ≥ 0.50

## Discussion

QoL is a multi-dimensional concept which reflects the overall functioning and well-being of the person. In this study, we compared the subjective QoL - mental and physical component among patients with schizophrenia spectrum disorders and MDD. We found that patients with schizophrenia generally reported better QoL than patients with MDD. Moreover, QoL differed across diagnostic groups with regards to the correlation with socio-demographics, such as age and ethnicity and clinical characteristics such as duration of mental illness, comorbid physical illness and severity of psychiatric symptoms.

Consistent with previous studies [43], compared to patients with MDD, patients with schizophrenia had poorer education, were more likely to be unemployed, have less income and were single. Moreover, patients with schizophrenia had longer duration of illness and a higher proportion of them were on medications. Depressive disorder, on the other hand, typically started later in life, perhaps when they were facing stress from marriage, raising kids or employment.

Patients with schizophrenia reported better mental and physical well-being than patients with MDD, suggesting possible explanations underlying these differences of subjective QoL. It is possible that patients with psychosis lacked self-awareness of their illness and social environment. Consequently, they may develop self-protective strategies and assign meaning to their lives, leading to reporting of better subjective QoL than patients with MDD [44]. On the other hand, patients with MDD suffer from symptoms, such as anhedonia, depressed mood, pessimistic outlook, decreased motivation and energy level and are therefore inclined to report worse QoL than patients with schizophrenia. Our finding is in contrast to another study that found no significant difference in subjective QoL between patients with schizophrenia and depressive disorder using the Quality of Life Satisfaction and Enjoyment Questionnaire (Q-LES-Q) [45]. This discrepancy could be due to the different instrument that researchers have utilized to measure subjective QoL. Moreover, despite controlling for several socio-demographics and clinical characteristics, the difference in self-reported QoL between the two clinical groups could still be attributed to other factors, such as previous history of hospitalizations and medication dosages, which we didn’t collect for this study.

Older patients with schizophrenia reported better scores of mental health than their younger counterparts after controlling for other socio-demographics and clinical characteristics. This has been similarly observed among the general population [46, 47], where it has been reported that older individuals reported higher levels of positive mental health than younger people. Increased spiritual involvement, personal characteristics and experiences of setbacks in life may explain this difference [48, 49]. However, no correlation was observed between age and mental health among patients with MDD, which was possibly due to the reason that patients with MDD are inclined to be negative about their life experiences which hampers their subjective well-being.

With regards to physical health, older patients with MDD reported lower scores of physical function than those who were younger while older patients with schizophrenia reported higher scores of physical function than their younger counterparts. The positive correlations of age with physical function among patients with schizophrenia may be related to the self-protective strategies adopted. Older patients with schizophrenia include those with relatively late onset schizophrenia [50]. Patients with late-onset schizophrenia tend to have a better premorbid functioning, fewer negative symptoms, less severe neurocognitive impairments [51], and require lower daily doses of antipsychotics than patients with early onset of disorder [52, 53]. This may explain our finding that older patients with schizophrenia tend to report better physical health than their younger counterparts.

Compared with Chinese patients with schizophrenia, those of Malay, Indian and other ethnicities reported lower scores of physical function. This was not observed among patients with MDD. The differences of physical function within the ethnic groups among patients with schizophrenia is similar to what has been previously reported among the general population, which could be associated with ethnic specific clinical risk factors, health behaviors and socio- cultural parameters [54]. It is possible that in patients with MDD, the overwhelming symptoms of lack of energy and low mood obscure these differences.

It is well recognized that among patients with schizophrenia, the severity of psychiatric symptoms is negatively associated with subject’s mental health and daily functioning [55]. In our study, compared with other symptoms, positive factor was correlated with impaired social functioning and the depressive factor was correlated with impaired mental health, vitality and general health to a larger extent. This suggests that social functioning in patients with schizophrenia may be more responsive to therapeutic interventions targeting positive symptoms and to improve subjective mental health, vitality and general health, we may need to focus more on the amelioration of depressive symptoms. On the other hand, improvement of various subdomains of QoL may also help to ameliorate the corresponding clinical psychiatric symptoms among patients with schizophrenia.

The depressive factor that we identified in patients with schizophrenia using PANSS score was similar to the depressive symptoms reported by patients with MDD using PHQ-8 questionnaire. Both of them contain a measurement of heterogeneous symptoms, including depression, anxiety and social avoidance. Among patients with MDD, most of the subdomains of mental well-being or physical well-being are highly correlated with depressive symptoms. The subjective QoL among patients with MDD, therefore, may be more responsive to therapeutics targeting core psychiatric symptoms than the patients with schizophrenia. Indeed, it has been reported that for patients with MDD, both the subjective QoL and clinical condition improved better during hospitalization than among patients with diagnosis of schizophrenia [44, 56].

## Conclusions

In summary, patients with schizophrenia spectrum disorders reported better QoL compared to patients with MDD. Patients’ socio-demographics and psychiatric symptoms were correlated with subjective QoL but differ in direction or the strength of correlation across diagnostic groups. Although this study is limited by the nature of cross-sectional study design and by a relatively small sample size, our findings highlight the need to assess the subjective QoL of patients with psychiatric symptoms seen clinically and their associations as this may allow for better optimization of the management of their illness. Future studies with more detailed information, such as the severity and duration of comorbid physical disease, medication history and dosage, non-pharmacological treatment history and the temporal relationship between the mental disorders and comorbid physical illness are needed for a more in-depth understanding of QoL of those with psychiatric illnesses.

## Data Availability

All data generated or analyzed in this study is included in this manuscript. The raw data are not publicly available due to them containing information that could compromise research participants’ privacy or consent, but may in part be available from the corresponding author on reasonable request.

## Abbreviations

ANCOVA: Analysis of covariance
BP: Bodily pain
DSM-IV: Diagnostic and Statistical Manual of Mental disorders, Fourth edition
GH: General health perceptions
MDD: Major depressive disorder
MH: General mental health
*Ƞ*^*2*^: Partial eta-squared
PANSS: Positive and Negative Syndrome Scale
PF: Physical functioning
PHQ-8: Eight-item Patient Health Questionnaire
Q-LES-Q: Quality of Life Satisfaction and Enjoyment Questionnaire
QoL: Quality of life
r: Correlation coefficients
RE: Role limitations due to personal or emotional problems
RP: Role limitations due to physical health problems
SD: Standard deviation
SF: Social functioning
SF-36: 36-item Short Form Survey Instrument
SGD: Singapore dollars
VT: Vitality
